# The Regulation and Double-Edged Roles of the Deubiquitinase OTUD5

**DOI:** 10.3390/cells12081161

**Published:** 2023-04-14

**Authors:** Lin Fu, Kun Lu, Qian Jiao, Xi Chen, Fengju Jia

**Affiliations:** 1 School of Basic Medicine, Qingdao University, Qingdao 266072, China; 2 School of Nursing, Qingdao University, Qingdao 266072, China

**Keywords:** OTUD5, immunity, DNA damage repair, tumors

## Abstract

OTUD5 (OTU Deubiquitinase 5) is a functional cysteine protease with deubiquitinase activity and is a member of the ovarian tumor protease (OTU) family. OTUD5 is involved in the deubiquitination of many key proteins in various cellular signaling pathways and plays an important role in maintaining normal human development and physiological functions. Its dysfunction can affect physiological processes, such as immunity and DNA damage repair, and it can even lead to tumors, inflammatory diseases and genetic disorders. Therefore, the regulation of OTUD5 activity and expression has become a hot topic of research. A comprehensive understanding of the regulatory mechanisms of OTUD5 and its use as a therapeutic target for diseases is of great value. Herein, we review the physiological processes and molecular mechanisms of OTUD5 regulation, outline the specific regulatory processes of OTUD5 activity and expression, and link OTUD5 to diseases from the perspective of studies on signaling pathways, molecular interactions, DNA damage repair and immune regulation, thus providing a theoretical basis for future studies.

## 1. Introduction

Ubiquitination is an important post-translational modification of proteins that affects the degradation and localization of modified proteins, thereby regulating physiological processes, such as cell proliferation, differentiation, apoptosis and DNA damage repair [1,2]. The ubiquitination of proteins is a reversible process. Ubiquitin ligase mediated ubiquitination modifications are antagonized by deubiquitinases (DUBs) [3]. The dynamic balance of intracellular ubiquitination and deubiquitination maintains the normal physiological functions of the body, but its imbalance can lead to a variety of diseases, such as cancer, inflammation and neurological disorders [2,4].

The deubiquitinating enzyme OTUD5, also known as deubiquitinating enzyme A (DUBA), is a member of the deubiquitinating enzyme OTU family. By deubiquitinating other proteins, OTUD5 is an important regulator of several physiological processes, such as immune signaling and DNA damage responses. The dysregulation of these processes can increase the risk of associated diseases, including inflammation, cancer and genetic disorders.

OTUD5 was originally identified as a negative regulator of type I interferon (IFN), and it functions in innate immunity [5]. In recent years, its importance in acquired immunity has also been gradually uncovered [6]. In line with this, many studies have suggested that OTUD5 may play a key role in the development and progression of immune disorders and inflammation. In addition, the function of OTUD5 in a variety of cancers is beginning to be understood, with its different levels of expression and different functions in different tumors. In hepatocellular carcinoma (HCC), non-small cell lung cancer (NSCLC) and cervical cancer, OTUD5 exerts oncogenic effects by removing the ubiquitination modifications of the tripartite motif containing 25 (TRIM25), tumor protein P53 (TP53), programmed cell death 5 (PDCD5) and phosphatase and tensin homolog (PTEN) [7,8,9]. In breast cancer and colon cancer, OTUD5 promotes carcinogenesis by regulating the mammalian target of rapamycin (mTOR) and Hippo signaling pathways [10,11,12].

In this review, we analyze the molecular basis of OTUD5 function, starting from the structural features of OTUD5. We review and analyze studies on its molecular mechanisms and functions in cellular processes (immune processes and DNA damage responses) and human diseases, including tumors, inflammation and genetic disorders. We also summarize the mechanisms that regulate the activity and expression of OTUD5. In addition, we discuss therapeutic opportunities and propose future research directions for OTUD5. A systematic review of its function and regulation suggests that OTUD5 is a potential therapeutic target for a variety of diseases.

## 2. Structural Characteristics of OTUD5

### 2.1. Ubiquitination and Deubiquitination Processes

Ubiquitin is a small-molecule protein that is widely distributed in eukaryotic cells and plays an important role in the cell by modifying associated proteins [13,14]. The process of ubiquitination refers to the covalent attachment of ubiquitin to target proteins in the form of single-site monoubiquitination, multi-site monoubiquitination, and polyubiquitin chains. Polyubiquitination modification refers to the ubiquitination of the seven lysine residues of ubiquitin (K6, K11, K27, K29, K33, K48 and K63) and its N-terminal methionine (Met1) to form an isopeptide-linked ubiquitin chain [15,16]. The process of ubiquitination involves a series of enzymatic reactions and usually requires the participation of three enzymes, including E1 ubiquitin-activating enzyme, E2 ubiquitin-conjugating enzyme and E3 ubiquitin ligase [17].

DUBs can remove ubiquitin chains or inhibit the catalytic function of ubiquitin-related enzymes, thereby inhibiting the ubiquitination process and regulating protein stability and function [18]. Deubiquitinating enzymes mainly include seven evolutionarily conserved superfamilies: the ubiquitin-specific proteases (USPs) superfamily, Machado-Joseph domain-containing proteases (MJDs) superfamily, OTU superfamily, JAMM/MPN domain-associated Zn-dependent metalloproteases (JAMMs) superfamily, ubiquitin C-terminal hydrolases (UCHs) superfamily, zinc finger-containing ubiquitin peptidase 1 (ZUP1) superfamily and ubiquitin-containing proteases (MINDYs) superfamily [19].

Based on the molecular structure of the protein, the OTUs superfamily can be divided into four subfamilies: the subfamily of proteins containing the ovarian tumor structural domain (OTUDs) (OTUD1, OTUD2/YOD1, OTUD3, OTUD4, OTUD5/DUBA, OTUD6A, OTUD6B and ALG13), the subfamily of ubiquitin aldehyde binding proteins (Otubains) containing the OTU structural domain (OTUB1 and OTUB2), the A20-like subfamily (A20, Cezanne2/OTUD7A, Cezanne/OTUD7B, TRABID/ZRANB1, VCPIP) and the OTU DUB with linear linkage specificity (OTULIN) [20,21].

### 2.2. Structural Characteristics of OTUD5

**Figure 1:**
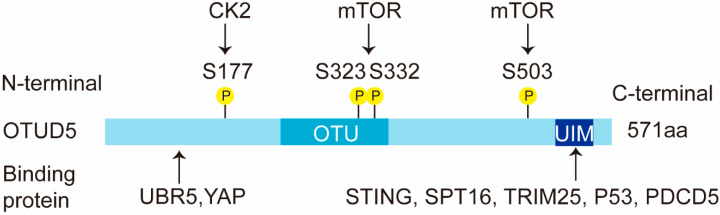
Schematic representation of the OTUD5 domains. OTUD5 has two structural domains, an N-terminal OTU domain and a C-terminal UIM domain. The human OTUD5 transcript is 571 aa. OTUD5 interacts with multiple proteins, such as YAP, SPT16, TRIM25, TP53 and PDCD5. This figure shows the diverse binding fragments of these proteins. OTUD5 is phosphorylated by kinase CK2 and mTOR. The phosphorylation sites are shown in the figure. P: phosphorylation.

OTUD5 is a cysteine protease and an important member of the OTU subfamily of deubiquitinating enzymes [5]. In humans, OTUD5 consists of 571 amino acids and is approximately 60 kD in size (Figure 1). It contains two conserved structural domains: the OTU structural domain (catalytic domain) and the ubiquitin-interacting motif (UIM) structural domain. The OTU domain of OTUD5 comprises about 120 amino acids, which is much smaller than the OTU domain of OTUBs or A20 OTUs. The integrity of the UIM domain is also particularly important for the function of OTUD5. The stimulator of interferon genes (STING), suppressor of ty 16 homolog (SPT16), TRIM25, TP53 and PDCD5 have all been reported to interact with the UIM structural domain of OTUD5 [7,22,23]. In addition, the N-terminal region of OTUD5 (1-212 aa) is required for its interaction with the ubiquitin protein ligase E3 component N-recognin 5 (UBR5) and the yes-associated protein (YAP) [12] (Figure 1).

The OTU family shows a preference for ubiquitin linkage types [24]. OTUD5 mainly prefers the K48 and K63 ubiquitin chain types [24]. K48-polyubiquitin chains and K63-polyubiquitin chains have been studied more extensively, with K48-polyubiquitin chains mainly playing a degradation role and K63-polyubiquitin chains usually not involved in degradation functions, but mainly performing signal transduction and DNA repair functions [15]. Unlike other deubiquitinating enzymes, the phosphorylation of OTUD5 at a single residue (Ser177) is required for the activation of the enzyme [25].

## 3. Physiological Processes Regulated by OTUD5

### 3.1. Immunity

Viruses pose a serious threat to human health. The host is able to defend itself against invading viruses through antiviral innate and adaptive immune responses, which provide a strong defense for the organism [26]. A rigorous and precise regulatory process of the immune system is the basis for effective pathogen clearance and the prevention of host overreaction [27]. The pathogen-associated molecular patterns (PAMPs) of viral recognition by the innate immune system depend on pattern recognition receptors (PRRs), including Toll-like receptors (TLRs), retinoic acid-inducible gene-I (RIG-I)-like receptors (RLRs) and DNA sensors. IFNs and pro-inflammatory cytokines produced by various signaling pathways can effectively limit viral spread and regulate the course of the subsequent adaptive immune response [28]. Through the regulation of different substrates, OTUD5 can play double-edged roles in immune response.

#### 3.1.1. Innate Immunity: The TLR and RLR Signaling Pathway

Innate immunity is the body’s first line of defense against pathogen invasion. IFN-I production is an important host defense mechanism induced by PRRs [29]. Studies have shown that OTUD5 is able to attenuate the IFN-I response induced by TLR and RLR signaling [5]. TLRs are the first known PRRs and are a central part of the innate immune system. TLRs are transmembrane proteins that recognize viral components in extracellular and post-phagocytic or endocytoplasmic vacuoles, subsequently activating TRAF3 and ultimately inducing IFN-I and pro-inflammatory cytokines to counteract viral invasion [30]. RLRs have three members: RIG-I, melanoma differentiation-associated gene 5 (MDA5) and laboratory of genetics and physiology 2 (LGP2). In the RLR signaling pathway, TRAF3 activation by RIG-I and MDA5 mediates the downstream signaling pathway by binding to TANK-binding kinase 1 (TBK1) [31]. Subsequently, TBK1 binds to IKK and then phosphorylates the transcription factor IRF3, which is required for the nuclear translocation of IRF3 and downstream gene activation.

**Figure 2:**
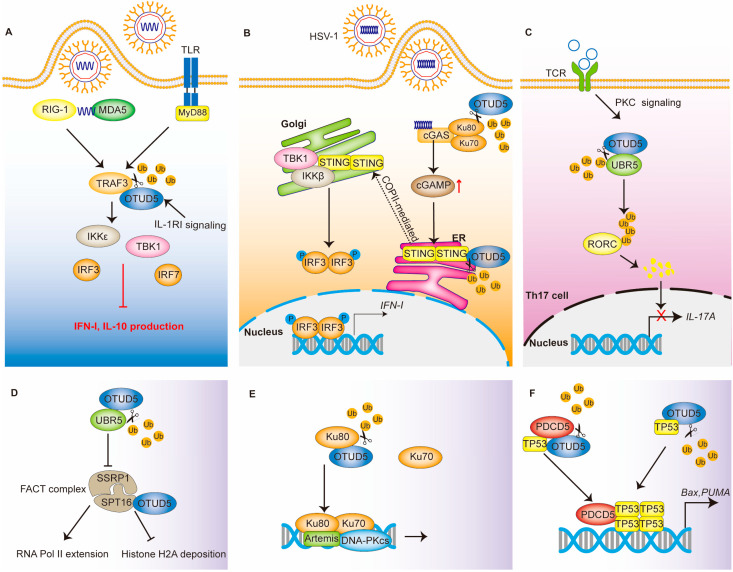
The regulatory network of OTUD5 in immune response and DNA damage repair. (**A**) OTUD5 in TLR and RLR signaling. OTUD5 cleaves the K63-linked polyubiquitin chain on TRAF3, leading to its dissociation from TBK1 and the inhibition of IFN-I production. (**B**) OTUD5 in the cGAS-STING signaling pathway. OTUD5 deubiquitinates and stabilizes the STING protein. STING then recruits and activates TBK1, which ultimately induces IFN- I production. (**C**) OTUD5 in adaptive immunity. OTUD5 deubiquitinates the E3 ligase UBR5, which ultimately leads to the inhibition of IL-17A production. (**D**–**F**) OTUD5 functions in DNA damage response by regulating the FACT complex, Ku80, PDCD5 and TP53. P: phosphate; Ub: ubiquitin.

Thus, TRAF3 plays important roles in both the TLR and RLR signaling pathways. Ubiquitination modifications can regulate the stability and activity of the TRAF3 protein. RNF166 binds and ubiquitinates TRAF3, thereby promoting the binding of TRAF3 to its downstream molecules and positively regulating its function [32]. The RNF216 ubiquitination of TRAF3 leads to its degradation [33]. As a deubiquitinating enzyme, OTUD5 is able to selectively cleave the K63-linked polyubiquitin chain on TRAF3, which leads to its dissociation from TBK1 complexes and the inhibition of the downstream signaling pathway [5] (Figure 2A).

In addition to PAMPs, PRRs also recognize damage-associated molecular patterns (DAMPs) released from damaged host cells and tissues. DAMPs include hyaluronic acid, histones, mRNAs, cholesterol crystals and others [34]. The specific function of OTUD5 in the body’s response to DAMPs remains unclear.

#### 3.1.2. Innate Immunity: The cGAS-STING Signaling Pathway

Upon the binding of DNA, cGAS begins to synthesize the second messenger cyclic GMP-AMP (cGAMP). Subsequently, cGAMP binds and activates the STING dimer. The activated STING is transported from the endoplasmic reticulum (ER) to the Golgi apparatus via COPII-mediated vesicles [35]. The activated STING recruits and activates TBK1 and IKKβ, which promote the nuclear import of IRF3, ultimately producing IFN-I [36].

OTUD5 interacts with STING, cleaves its K48-linked polyubiquitin chain and promotes its stability (Figure 2B) [37]. The stabilization of STING promotes an antiviral IFN-I response, which effectively controls damage caused by DNA viruses, such as the herpes simplex virus type 1 (HSV-1). The knockdown of OTUD5 results in an accelerated turnover of STING and, subsequently, impaired IFN-I signaling [37]. Functionally acquired mutations in STING can lead to a severe autoinflammatory disease known as STING-associated vasculopathy with its onset in infancy (SAVI) [38,39]. In vivo experiments have also shown that myeloid cell and dendritic cell (DC)-conditional Otud5 knockout mice are more susceptible to HSV-1 infection than the corresponding control mice [37]. Interestingly, mice with a knockout of OTUD5 developed melanoma more rapidly. Therefore, the interaction of OTUD5 in tumor and immunity deserves further investigation.

#### 3.1.3. Acquired Immunity

OTUD5 is also important in T cells. It was shown that the Ser177 site-phosphorylated OTUD5 protein was increased in response to T-cell antigen receptor (TCR) stimulation. This suggests that OTUD5 also plays a role in the process of acquired immunity [6]. T helper type 17 (TH17) cells, which produce interleukin-17A (IL-17A) and IL-17F, have been implicated in the pathogenesis of several autoimmune diseases [40]. OTUD5, which accumulates in activated T cells, is able to stabilize UBR5 in response to TGF-β signaling [6]. Then, the E3 ligase UBR5 is able to ubiquitinate RAR-related orphan receptor C (RORC) and lead to its degradation. Notably, RORC is a transcription factor of IL-17A [41]. Thus, OTUD5 regulates the production of IL-17 by T cells, and through this, it functions as a negative regulator in the process of acquired immunity [22] (Figure 2C).

### 3.2. DNA Damage

#### 3.2.1. FACT Complex

An organism is able to maintain genomic and transcriptome integrity in response to genotoxic stress through a variety of mechanisms [42]. One of these mechanisms is the rapid transcriptional cessation at or near the DNA lesion. Transcriptional cessation can help DNA repair mechanisms access the lesion site and can allow the repair process to proceed. Once the DNA is well repaired, the transcription process resumes [43]. The heterodimer structure-specific recognition protein 1(SSRP1) and SPT16 form the chromatin transcription elongation factor-facilitates chromatin transcription (FACT) [44]. FACT is capable of interacting specifically with histone H2A/H2B to affect nucleasome disassembly and transcription elongation [45]. UBR5 can inhibit the FACT histone chaperone complex, which would result in the arrest of RNA Pol II elongation at DNA double-strand break (DSB) lesions [46]. OTUD5 localizes to DSBs and interacts with UBR5. By removing the ubiquitination of UBR5, it stabilizes UBR5 and inhibits RNA Pol II extension and RNA synthesis [23]. In addition, OTUD5 can interact with the SPT16 protein in the FACT complex and antagonize the deposition of histone H2A upon DSB damage by acting as a scaffold [23,47] (Figure 2D).

#### 3.2.2. Ku Heterodimers

Mammalian cells mainly use two distinct and complementary pathways—non-homologous end joining (NHEJ) and homologous recombination (HR) to repair DSBs, thereby maintaining genomic stability and eliminating oncogenic DNA lesions [48]. Ku heterodimers (Ku70/Ku80) have been shown to play an important role in NHEJ repair [49]. Ubiquitination of Ku80 can lead to the removal of Ku heterodimers from DSB sites while contributing to the selection of DSB repair pathways [50,51]. OTUD5 promotes NHEJ repair by increasing the stability of Ku80 [52] (Figure 2E). Cells in which OTUD5 is knocked out show excessive end excision. Meanwhile, in the S/G2 phase, OTUD5 knockdown promotes HR repair. Direct competition between Ku70/Ku80 heterodimers and MRN/CtIP for DSB binding affects the balance between HR and NHEJ repair [53]. OTUD5 stabilizes Ku80 and increases its accumulation at the DSBs [52]. Interestingly, Ku proteins, as co-receptors, also promote the formation of cGAS-binding DNA and cGAS multimers, further enhancing the enzymatic activity of cGAS and thus positively regulating the activation of the cGAS-STING pathway [54]. In the innate immune response, OTUD5 can promote the stability of STING [37]. The function of OTUD5 in the interaction between DNA damage and innate immunity needs to be further investigated.

#### 3.2.3. TP53 and PDCD5

TP53 is a tumor suppressor and transcription factor that regulates cell division; it prevents cells with mutated or damaged DNA from dividing and inducing apoptosis [55]. OTUD5 can bind to TP53 and regulate its ubiquitination levels [56]. Thus, in response to DNA damage stress, OTUD5 is essential for the rapid activation of TP53-dependent transcription and apoptosis [56].

PDCD5 positively regulates TP53-mediated apoptosis and accumulates rapidly after DNA damage [57]. Under genotoxic stress, OTUD5 binds to PDCD5 and increases PDCD5 stability by mediating the deubiquitination of PDCD5 [22]. Accordingly, OTUD5 overexpression has been shown to effectively enhance the activation of PDCD5 and TP53. The sequential activation of PDCD5 and TP53 have also been abolished after OTUD5 knockdown. Thus, the OTUD5-dependent stabilization of PDCD5 is required for the sequential activation of TP53 in response to DNA damage [22] (Figure 2F).

## 4. OTUD5-Related Diseases

### 4.1. Tumors

OTUD5 expression has been reported to be significantly upregulated in bladder cancer [11] and downregulated in HCC, cervical cancer and NSCLC [7,58]. OTUD5 is involved in the tumorigenesis and progression of several common cancers through the deubiquitination and stabilization of some key proteins or enzymes. However, the effect of OTUD5 on cancer development and progression is not fixed. OTUD5 not only promotes cancer development, but also inhibits the development of several cancers. The specific function of OTUD5 in different cancers needs to be analyzed on a case-to-case basis.

#### 4.1.1. Tumor Promoter

##### mTOR Pathway

**Figure 3:**
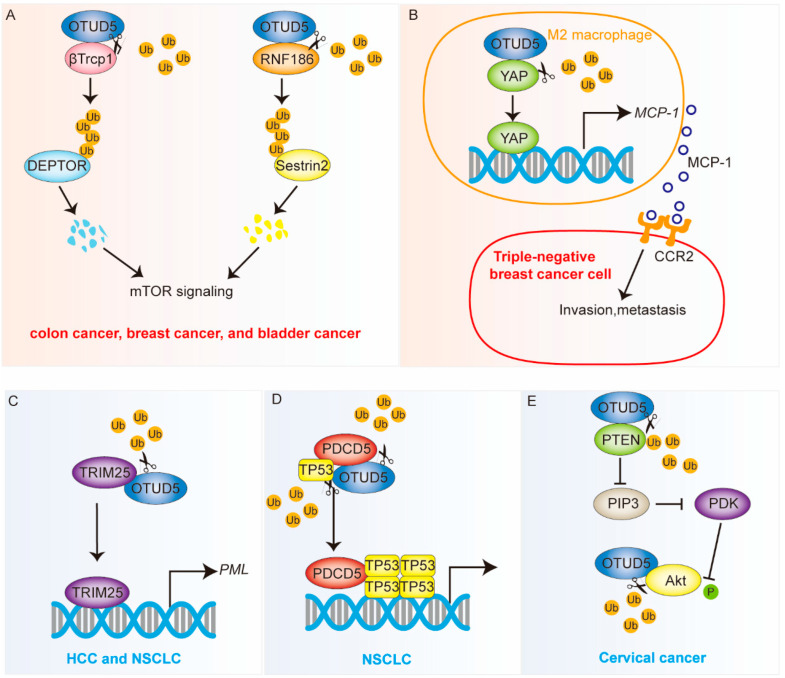
OTUD5 is involved in the development of cancer (see also Table 1). OTUD5 plays dual roles in tumor progression. OTUD5 is an oncogene in colon cancer, breast cancer and bladder cancer but a tumor suppressor in HCC, NSCLC and cervical cancer. OTUD5 affects multiple signaling pathways, including the mTOR, Hippo and Akt pathway. OTUD5 stabilizes key proteins, such as βTrcp1, Sestrin2, YAP, TP53, PDCD5 and Akt. OTUD5 regulates the transcriptional activity of TRIM25. (**A**,**B**) The orange background represents the cancer-promoting function. (**C**–**E**) The blue background represents the cancer-suppressing function. Ub: ubiquitin.

The mTOR pathway regulates a variety of physiological processes, including cell growth and cancer progression [59]. OTUD5 stabilizes the β-transducin repeat-containing protein 1 (β-TrCP1) through its deubiquitinase activity, leading to the degradation of the DEP domain-containing mTOR-interacting protein (DEPTOR) [10] (Figure 3A). DEPTOR is a repressor protein of mTOR [60]. Thus, OTUD5 can regulate gene expression downstream of mTOR. The knockdown of OTUD5 has caused several mTOR-related phenotypes, such as a decrease in cell size and an increase in autophagy in mammalian cells. In addition, OTUD5 knockdown has been shown to inhibit the proliferation of cancer cell lines with mutations that activate the mTOR pathway; these include HT29 colon cancer cells and MCF7 breast cancer cells [10].

The ubiquitin ligase ring finger protein 186 (RNF186) can ubiquitinate and modify Sestrin2, which ultimately leads to its degradation [61]; Sestrin2 is also an inhibitor of the mTOR pathway [62]. The deubiquitinase OTUD5 regulates mTOR signaling and promotes bladder cancer progression (Figure 3A). The specific mechanism is that OTUD5 deubiquitinates RNF186 and stabilizes its protein levels, which, in turn, leads to the increased degradation of Sestrin2 and the subsequent activation of the mTOR pathway [11].

Interestingly, OTUD5 positively regulates the mTOR pathway by removing ubiquitin molecules from the ubiquitin ligases β-TrCP1 and RNF186, thereby stabilizing both proteins. As a deubiquitinating enzyme, OTUD5 can also be modified by ubiquitin ligases [63]. The relationship between OTUD5 and ubiquitin ligases under different spatiotemporal conditions needs to be further investigated.

##### Hippo Pathway

YAP is a downstream transcription factor of the Hippo pathway, and its role in tumor immunity has become increasingly apparent in recent years [64]. Macrophages are highly plastic and can be polarized into M1 or M2 subtypes, depending on various signals in the complex microenvironment, thereby influencing tumor development [65]. Zhang et al. found that the treatment of triple-negative breast cancer (TNBC) cells upregulated YAP expression in macrophages. The specific mechanism was that OTUD5 was able to remove the ubiquitination modification of YAP, thereby stabilizing its protein level [12] (Figure 3B). The high expression of YAP in TNBC polarizes macrophages to an M2-like phenotype and subsequently promotes the metastasis of TNBC cells via the monocyte chemoattractant protein-1 (MCP-1)/C-C motif chemokine receptor 2 (CCR2) pathway [12]. Therefore, OTUD5 may act as a promoter of TNBC metastasis by stabilizing the YAP protein.

#### 4.1.2. Tumor Inhibitor

##### TRIM25

An analysis of clinical samples revealed that OTUD5 expression is significantly downregulated in hepatocellular carcinoma and non-small cell lung cancer. Furthermore, reduced OTUD5 levels have been associated with poor clinical outcomes in liver cancer and NSCLC patients [7]. OTUD5 has been shown to reduce the ubiquitination level of TRIM25, resulting in its decreased transcriptional activity. TRIM25, a member of the TRIM family, can regulate cell proliferation and migration in an E3 ubiquitin ligase-dependent or non-dependent manner [66,67,68]. For example, TRIM25 can act as a transcriptional regulator to regulate the expression of breast cancer metastasis-associated genes [69].

By reducing the ubiquitination level of TRIM25, OTUD5 suppresses promyelocytic leukemia protein (PML) expression [7] (Figure 3C). PML, also known as tripartite motif containing 19 (TRIM19), is capable of forming large nucleosomes called PML nuclear bodies (PML-NBs), which are present in almost all human cell types and exhibit macromolecular spherical structures [70]. Thus, OTUD5 knockdown was able to accelerate tumor growth in a nude mouse model.

##### TP53 and PDCD5

*TP53* is the most commonly mutated gene and a classical tumor suppressor [71]. PDCD5 positively regulates TP53-mediated apoptosis [57]. OTUD5 can increase the stability of TP53 and PDCD5 through deubiquitination [22] (Figure 3D). Studies have shown that the downregulation of OTUD5 in NSCLC tissues is significantly associated with poor prognosis. OTUD5 knockdown has been shown to cause TP53 and PDCD5 inactivation and promote the proliferation and metastasis of NSCLC cells while inhibiting their apoptosis. In addition, OTUD5 knockdown also enhances the resistance of NSCLC cells to doxorubicin and cisplatin [8].

##### PTEN and Akt

PTEN is a tumor suppressor with phosphatase activity that inhibits the development of many tumors [72]. Li et al. found that OTUD5 stabilizes PTEN through deubiquitination and that the overexpression of OTUD5 inhibits NSCLC cell proliferation, invasion and migration [9] (Figure 3E). In cervical cancer, OTUD5 also plays an important oncogenic function. The low expression of OTUD5 is associated with a poor prognosis for cervical cancer [58]. In addition, the overexpressed OTUD5 cells are highly sensitive to radiotherapy [73]. PTEN could inhibit Akt activity by dephosphorylating PIP3 [74]. OTUD5 overexpression has been shown to significantly downregulate the phosphorylation level of Akt in cervical cancer cells. Interestingly, OTUD5 overexpression also significantly downregulated the ubiquitination level of Akt in cervical cancer cells, suggesting that OTUD5 may be a deubiquitinating enzyme for Akt [73] (Table 1).

**Table 1:** Expression and clinical significance of OTUD5 in various cancers.

Target Substrate	Tumor Type	Result of Deubiquitination	Affected Pathway or Event	Effect	References
βTrCP1	Colon cancer Breast cancer	Stable protein	mTOR pathway	Enhanced cancer cell proliferation	[10]
RNF186	Bladder cancer	Stable protein	mTOR pathway	Enhanced cancer cell progression	[11]
YAP	Triple-negative breast cancer	Stable protein	Hippo pathway	Enhanced cancer cell metastasis	[12]
TRIM25	Hepatocellular carcinoma and non-small cell lung cancer	Decreased transcriptional activity	-	Reduced tumor growth	[7]
P53/PDCD5	Non-small cell lung cancer	Stable protein	Apoptosis	Reduced cancer cell proliferation and metastasis	[8]
PTEN	Non-small cell lung cancer	Stable protein	Akt signaling	Inhibited proliferation, invasion and migration	[9]
Akt	Cervical cancer	Stable protein	Akt signaling	Sensitive to radiotherapy	[73]

### 4.2. Inflammation in the Digestive System

OTUD5 regulates both innate and adaptive immune regulatory processes, so it is not surprising that its dysfunction would trigger inflammatory responses. The immune response associated with inflammatory bowel disease (IBD) is characterized by the overproduction of multiple inflammatory cytokines, which are thought to perpetuate and amplify the pathological process [75]. In contrast, OTUD5 is able to regulate cytokine production through innate and adaptive immune cells [5,6,37]. Crohn’s disease (CD) and ulcerative colitis (UC) are two common IBDs [76]. Compared to controls, the expression of the OTUD5 protein was increased in inflammatory ileal and colonic mucosal samples from patients with CD and UC, but not at the RNA level [77]. Consistent with this is stimulation with the inflammatory cytokine IFN-γ, which resulted in the significant upregulation of OTUD5 expression in lamina propria mononuclear cells (LPMC). Furthermore, OTUD5 overexpression resulted in a significant decrease in TNF-α RNA in LPMC, whereas the levels of IL-6 and IL-10 were unchanged [77].

Genome-wide association studies (GWAS) in primary biliary cholangitis (PBC) have identified variants associated with the disease on the X chromosome. A population-specific meta-analysis revealed a significant genome-wide locus in the east Asian population pointing to the same region (rs7059064), which included an enhancer (GH0XJ048933 within OTUD5) [78]. FOXP3 is a major T regulatory cell lineage-specific factor [79]. Asselta et al. predicted that GH0XJ048933 could also target FOXP3. Moreover, both OTUD5 and FOXP3 RNAs were found to be upregulated in PBC cases [78]. This suggests the possibility that OTUD5 may have a function in PBC, the exact mechanism of which requires further investigation.

Altered immune homeostasis and T cell involvement are present in chronic pancreatitis (CP) [80]. The exposure of BTB and CNC homologous 2(Bach2)-silenced CD4^+^ T lymphocytes to chronic pancreatitis tissue extracts leads to a decrease in OTUD5 and increased Th17 cell differentiation. This suggests a potential regulatory role for OTUD5 in advanced CP [81]. Whether the transcription factor Bach2 directly regulates the expression of OTUD5 needs to be determined.

### 4.3. Genetic Diseases

Genetic disorders are diseases mainly caused by defects in DNA. Most hereditary diseases currently have no effective treatment, and some of them are severe, with high mortality and disability rates, and thus the prevention of hereditary diseases is particularly important [82]. Furthermore, it is critical to determine the genetic causes of diseases using the latest omics methods for disease prevention [83,84].

Tripolszki et al. identified 13 males in a family with an X-linked syndrome due to a unique mutation in the *OTUD5* gene (c.598G > A). Patients with this syndrome may show growth retardation and ventricular enlargement before birth. Congenital heart disease, hypospadias and neurodevelopmental delay occur in the neonatal period. In infancy, patients with this mutation often die from sepsis. It is worth mentioning that female carriers of mutant genes are asymptomatic [85].

Beck et al. described a dysmorphic genetic disorder characterized by developmental delay and abnormalities of the brain, heart and facial features [86]. Sequencing revealed that these disorders are also caused by mutations in OTUD5, and thus these disorders were named linkage-specific deubiquitylation deficiency-induced embryonic defects (LINKED) syndromes. The processes of ubiquitination and deubiquitination are crucial in the development of the nervous system, and abnormalities in these processes will lead to the occurrence of nervous system-related diseases [14,87,88]. OTUD5 acts on protein substrates called chromatin remodelers, which include the AT-rich interaction domain 1A(ARID1A) and ARID1B, histone deacetylase 2 (HDAC2), host cell factor C1 (HCF1) and UBR5 [86]. These proteins play important roles in coordinating chromatin remodeling at central nervous system precursors and neural crest enhancers [46,89,90,91]. UBR5 is the only previously identified OTUD5 substrate among these candidates [23]. When OTUD5 is mutated, OTUD5 cannot remove the K48-ubiquitin chains and prevent the degradation of these chromatin regulators, thus leading to the abnormal development of neural precursors and neural cells [86]. OTUD5 has been reported to remove the K63-type ubiquitination modifications of TRIM25 and TRAF3 [5,7]. TRAF3 is also required for neural regeneration [92]. Whether OTUD5 exerts its function during embryonic development with selectivity for the ubiquitin chain form needs further confirmation.

In fact, life expectancy in LINKED syndrome may be longer than previously thought. Saida et al. described three patients with in vivo OTUD5 missense variants associated with LINKED syndrome [93]. They identified three males with X-linked intellectual disability (XLID) in two families with developmental delay. They shared clinical features, including hypotonia, short stature and unique facial features, such as hyperopia and nasal depression. These included a pair of adult siblings with a novel missense OTUD5 variant. They had a relatively mild phenotype, showed no cardiac or urogenital malformations and lived into their 40s [93]. The p.Arg404Trp variant was previously reported by Beck et al. to be a lethal variant of OTUD5 with a typical LINKED syndrome phenotype [86]. However, the case identified by Saida et al. is currently alive [93] (Table 2).

With regard to the pathogenic mutations, Beck et al. confirmed that these mutations affected the expression level, nuclear localization and ubiquitination enzyme activity of the OTUD5 protein, especially the K48-ubiquitin chain cleavage activity. The effects of the mutation on OTUD5 function were not investigated in depth by the other two study groups. Notably, the pathogenic variants (p.Asp256Asn, p.Arg274Trp and p.Asn293Ile) were found to be located in the OTU domain and may thus be related to their enzyme activity.

## 5. Regulation of OTUD5 Activity and Expression

OTUD5 plays important roles in tumors and immune regulation, and its expression varies among cells. Therefore, it is important to summarize the regulatory mechanisms of OTUD5 activity and expression to better understand OTUD5-related biological networks in human diseases.

### 5.1. Transcription Level Regulation

**Figure 4:**
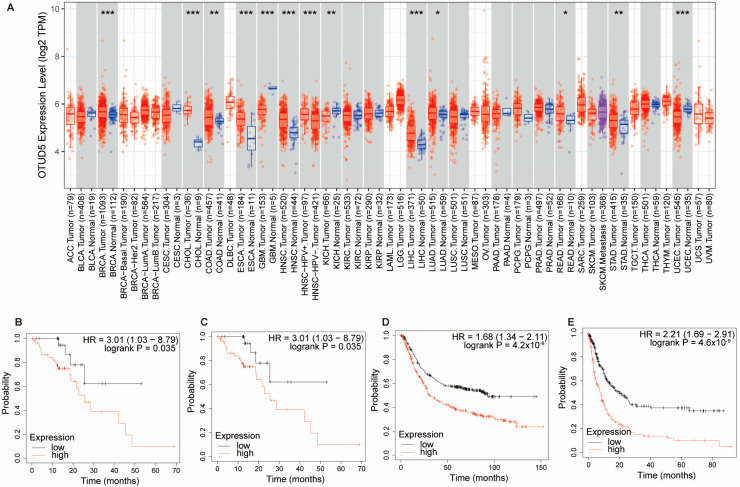
OTUD5 is aberrantly expressed in several types of tumors. (**A**) By analyzing the data in the TCGA database, we found that OTUD5 expression was significantly lower in glioblastoma (GBM), kidney chromophobe (KICH) and uterine corpus endometrial carcinoma (UCEC) and significantly highly expressed in breast cancer (BRCA), bile duct cancer (CHOL), colon cancer (COAD), esophageal cancer (ESCA), head and neck cancer (HNSC), liver cancer (LIHC), lung adenocarcinoma (LUAD), rectal cancer (READ) and stomach cancer (STAD). Data were analyzed using TIMER2.0. *p* < 0.05 *, *p* < 0.01 **, *p* < 0.001 ***. (**B**) The overall survival (OS) of OTUD5 in esophageal cancer (ESCA). (**C**) The recurrence-free survival (RFS) of OTUD5 in esophageal cancer (ESCA). High mRNA expression of OTUD5 was significantly associated with poor OS and RFS in ESCA. (**D**,**E**) The OS and progression-free survival (PFS) of OTUD5 in stomach cancer (STAD). High mRNA expression of OTUD5 was significantly associated with OS and PFS in STAD. (**B**–**E**) Data were analyzed using the Kaplan–Meier Plotter, a web-based survival analysis tool.

OTUD5 is aberrantly expressed in cancer and is considered to be a tumor suppressor or a tumor promoter in various types of cancer. OTUD5 expression has been reported to be significantly upregulated in bladder cancer [11] and downregulated in HCC, cervical cancer and NSCLC [7,58]. In NSCLC, mir-652 was able to target and inhibit OTUD5 expression, thereby promoting cell proliferation, invasion and migration [9]. In addition, mir-137, mir-144, mir-607, mir-937, mir-1913 and mir-3149 could inhibit the expression of OTUD5 in cervical cancer [58]. We also analyzed OTUD5 expression in a variety of tumors in the TCGA dataset (Figure 4A) [94]. Interestingly, the Kaplan–Meier survival analysis showed that the high mRNA expression of OTUD5 was significantly associated with poor overall survival in esophageal squamous cell carcinoma and gastric cancer (Figure 4B–E) [95]. The expression and function of OTUD5 in these two tumor types are currently unknown and need to be further investigated.

OTUD5 plays an important role in immune regulation [5,6,37]. The stimulation of bone marrow-derived dendritic cells (BMDCs) from WT and Il1r1^−/−^ mice with CpG, a ligand for TLRs, showed that CpG induced higher levels of the OTUD5 protein in Il1r1^−/−^ compared to WT BMDC. In the absence of IL-1RI signaling, the expression level of OTUD5 increases. Thus, IL-1RI signaling promotes TLR-dependent IFN-I production [96]. In CD4^+^ T lymphocytes from chronic pancreatitis tissues, the knockdown of the transcription factor Bach2 downregulates OTUD5 expression [81]. Whether Bach2 directly regulates the transcription of OTUD5 remains to be determined.

During the immune regulation process, some factors also feedback regulate the expression of OTUD5. It has been found that IFN-γ stimulation increases OTUD5 expression in LPMC from patients with inflammatory bowel disease. The specific mechanism may be that IFN-γ increases the phosphorylation of p38/MAPK [77]. Thus, it is possible that OTUD5 is also involved in the amplification of abnormal cytokine responses in inflammatory bowel disease.

### 5.2. Post-Translational Modifications

The post-translational modifications of proteins, including processes such as phosphorylation and ubiquitination, can affect protein activity and expression (Figure 1) [97]. Huang et al. reported the crystal structure of the pSer177 OTUD5–Ub complex. The phosphorylation of OTUD5 at Ser177 is required for the activation of the enzyme. A nuclear magnetic resonance (NMR) analysis revealed that the phosphorylation of Ser177 alone does not induce significant structural changes, and only after binding ubiquitin does Ser177-phosphorylated OTUD5 show significant structural changes that activate its enzymatic activity. Phosphate is essential for the recognition of ubiquitin molecules by OTUD5. Phosphorylation at this site is dependent on the kinase CK2 [25]. This phosphorylation regulates the conformational dynamics of the α1 helix and adjacent regions, thereby facilitating the folding of OTUD5 near its substrates [98].

mTOR is a serine/threonine protein kinase [99]. It directly phosphorylates the Ser323/332/503 sites of OTUD5 and activates its DUB activity. Furthermore, the activated OTUD5 removes the ubiquitination modification of βTrCP1, leading to the degradation of the mTOR inhibitory protein DEPTOR, which ultimately triggers the activation of the mTOR signaling pathway [10]. Thus, a positive feedback regulatory process is established between OTUD5 and mTOR signaling.

In response to TCR stimulation, the protein levels of the Ser177-phosphorylated OTUD5 were found to be upregulated. The protein kinase C (PKC) signaling pathway downstream of the TCR may post-translationally regulate the expression of OTUD5. The proteasome inhibitor MG-132 was also found to increase the protein level of OTUD5 in T cells, suggesting that OTUD5 is degraded by the proteasome in the absence of TCR stimulation [6].

RNF146 is thought to be a PARylation-dependent E3 ligase, and the PARylation of substrates by tankyrase 1 and 2 (TNKS1/2) is required for the activation of RNF146 E3 ubiquitin ligase catalytic activity [100]. By targeting and degrading several tumor suppressors, RNF146 can activate signaling pathways, such as Wnt and Hippo, thereby exerting pro-cancer effects [101,102]. Nie et al. found that RNF146 can ubiquitinate OTUD5 and lead to its degradation by the proteasome [63]. This is the only E3 ligase that has been reported for OTUD5. To enrich the regulatory network of OTUD5, we predicted the E3 ligases that could modify OTUD5 with the use of Ubibrowser 2.0 (Table 3) [103]. Among them, Smad ubiquitination regulatory factor 1 (SMURF1) and SMURF2 were found to play important roles in the development of several tumors [14]. The ability of these ubiquitin ligases to regulate OTUD5 needs to be further investigated.

## 6. Concluding Remarks and Future Perspectives

OTUD5 regulates the TLR, RLR and cGAS-STING signaling pathway in innate immunity [5,6,37]. OTUD5 can also reduce the IL-17 production by T cells [6]. The ability of OTUD5 to regulate innate and acquired immune processes implies a potential role for OTUD5 in the tumor microenvironment. The tumor microenvironment is characterized by nutrient competition and hypoxia [104,105]. This can lead to the immunosuppression or tolerance of immune cells, prompting immune cells to rely more on anaerobic respiration in order to meet energy metabolic requirements. These include accelerated effector T cells that are exhausted to increase immune checkpoint expression and the promotion of M2-like macrophage differentiation and accumulation [106,107]. OTUD5 is able to promote macrophage differentiation toward the M2 type by stabilizing the protein levels of YAP [12]. The activation of cGAS also plays a key role in antitumor immunity [108]. Mice lacking cGAS or STING has been shown to be more prone to tumor growth than controls, and in some of these models, their response to immune checkpoint inhibitor therapy was impaired [109,110]. The specific functions and regulatory networks of OTUD5 in different tumor microenvironments remain unclear and still need to be further explored.

Dynamic interactions between different cell populations can directly or indirectly promote competition for limited resources and space within the tissue [111]. Tumor growth requires the elimination of surrounding cells to facilitate its own growth. Tumor cells crowd out the surrounding normal cells through mechanical competitive pressure, which activates the NF-kB pathway of immune response and induces the apoptosis of surrounding normal cells, thus promoting tumor growth [112]. OTUD5 is capable of promoting P53-induced apoptosis in NSCLC cells [8], and its function in peritumor cells remains to be further investigated.

## Figures and Tables

**Table 2:** Clinical features of patients with OTUD5 mutations.

Numbers	Clinical Manifestation	OTUD5 Variant	Protein Change	Age	Status	References
*n* = 13	Neurodevelopmental delay, hydrocephalus and early lethality.	598G > A	Glu200Lys	4 days–37 y	12 deceased (from infancy (4 days–2 y), 6 y, 37 y) and 1 alive	[85]
*n* = 10	Global developmental delay with brain malformations, hirsutism, genitourinary defects and early lethality.	482_490del, 766G > A, 820C > T, 1055 T > C, 1210C > T, 1480 G > A	161_164del, Asp256Asn, Arg274Trp, Leu352Pro, Arg404Trp, Gly494Ser	2–14 y	4 deceased (from infancy (1–13 m), 1 deceased in utero) and 6 alive	[86]
*n* = 3	Severe short stature refractory epilepsy and congenital anomalies.	878A > T 1210C > T	Asn293Ile Arg404Trp	2–49 y	Alive	[93]

**Table 3:** Predicted E3s of OTUD5. (The UbiBrowser is a prediction and presentation website for E3/DUB-substrate interactions. By inputting the DUB of interest, the website will give the predicted substrate information and score.).

Gene Symbol (E3)	Domain_ Likelihood Ratio	Go_ Likelihood Ratio	Network_ Likelihood Ratio	Motif_ Likelihood Ratio	Confidence Score
SMURF2	1	3.63	3.83	4.09	0.853
NEDD4	1	2.78	3.83	4.09	0.837
MARCHF7	1	1	3.83	9.23	0.825
SMURF1	1	8.56	1	4.09	0.824
UBE4B	1	3.63	3.83	2.28	0.818
ITCH	1	1.99	3.83	4.09	0.817
SYTL4	1	8.56	1	3.35	0.811

## Data Availability

All data relevant to this review are included in the text, references, tables and figures.

## Abbreviations

OTUD5	OTU deubiquitinase 5
OTU	Ovarian tumor proteases
DUBA	Deubiquitinating enzyme A
DUBs	Deubiquitinases
IFN	Interferon
HCC	Hepatocellular carcinoma
NSCLC	Non-small cell lung cancer
TRIM25	Tripartite motif containing 25
TP53	Tumor protein P53
PDCD5	Programmed cell death 5
PTEN	Phosphatase and tensin homolog
mTOR	Mammalian target of rapamycin
USPs	Ubiquitin-specific proteases
MJDs	Machado-Joseph domain-containing proteases
JAMMs	JAMM/MPN domain-associated Zn-dependent metalloproteases
UCHs	Ubiquitin C-terminal hydrolases
ZUP1	Zinc finger-containing ubiquitin peptidase 1
MINDYs	Ubiquitin-containing proteases
UIM	Ubiquitin-interacting motif
STING	Stimulator of interferon genes
SPT16	Suppressor of Ty 16 Homolog
YAP	Yes-associated protein
PAMPs	Pathogen-associated molecular patterns
PRRs	Pattern recognition receptors
TLRs	Toll-like receptors
RIG-I	Retinoic acid-inducible gene-I
RLRs	RIG-I-like receptors
MDA5	Melanoma differentiation-associated gene 5
LGP2	Laboratory of genetics and physiology 2
TBK1	TANK-binding kinase 1
cGAMP	Cyclic GMP-AMP
ER	Endoplasmic reticulum
HSV-1	Herpes simplex virus-1
DC	Dendritic cell
SAVI	STING-associated vasculopathy with onset in infancy
TCR	T cell a ntigen receptor
TH17	T helper type 17
IL-17A	Interleukin-17A
RORC	RAR related orphan receptor C
FACT	Facilitates Chromatin Transcription
DSB	DNA double-strand break
SSRP1	Structure-Specific Recognition Protein 1
NHEJ	Non-homologous end joining
HR	Homologous recombination
βTrCP1	β-transducin repeat-containing protein 1
DEPTOR	DEP Domain-Containing MTOR-Interacting Protein
MCP-1	Monocyte chemoattractant protein-1
CCR2	C-C Motif chemokine receptor 2
PML	Promyelocytic leukemia protein
TRIM19	Tripartite motif containing 19
NBs	Nucleosomes
IBD	Inflammatory bowel disease
CD	Crohn’s disease
UC	Ulcerative colitis
LPMC	Lamina propria mononuclear cells
GWAS	Genome-wide association studies
PBC	Primary biliary cholangitis
CP	Chronic pancreatitis
Bach2	BTB and CNC homologous 2
LINKED	Linkage-specific deubiquitylation deficiency-induced embryonic defects
HDAC2	Histone deacetylase 2
XLID	X-linked intellectual disability
NMR	Nuclear magnetic resonance
PKC	Protein kinase C
TNKS1/2	Tankyrase 1 and 2
SMURF1	Smad ubiquitination regulatory factor 1
